# Nutritional Value, Fermentation Characteristics and In Vitro Degradability of Whole Wheat Hay Harvested at Three Stages of Maturity

**DOI:** 10.3390/ani12111466

**Published:** 2022-06-05

**Authors:** Xiaochen Lang, Meng Yang, Atef M. Saleem, Xiaojing Zhao, Hua Xu, Yan Li, Ruiting Xu, Jiaqiu Cao, Congcong Xu, Yushan Cui, Jia Li, Jiahui Li, Yizhao Shen, Yunqi Li, Jianguo Li, Yanxia Gao

**Affiliations:** 1 College of Animal Science and Technology, Hebei Agricultural University, Baoding 071001, China; lxc18703240436@163.com (X.L.); yangmeng6559@163.com (M.Y.); xuruiting5@163.com (R.X.); cjq19980920@163.com (J.C.); ccx202205@126.com (C.X.); cys15373197919@126.com (Y.C.); lijia961123@163.com (J.L.); lijiahui0209@126.com (J.L.); li-yunqi@163.com (Y.L.); jgli@hebau.edu.cn (J.L.); 2 Department of Animal and Poultry Production, South Valley University, Qena 83523, Egypt; atefmsaleem@yahoo.com; 3 Baoding Vocational and Technical College, Baoding 071000, China; zxj2002@126.com; 4 Langfang Animal Husbandry Station, Langfang 065000, China; xuhua78@163.com; 5 College of Veterinary Medicine, Hebei Agricultural University, Veterinary Biological Technology Innovation Center of Hebei Province, Baoding 071001, China; dyly@hebau.edu.cn; 6 Hebei Technology Innovation Center of Cattle and Sheep Embryo, Baoding 071001, China; 7 Hebei Research Institute of Dairy Industry Technology, Shijiazhuang 050221, China

**Keywords:** whole wheat hay, in vitro gas production, maturation stages, nutritional value assessment

## Abstract

**Simple Summary:**

Although wheat is primarily planted to harvest wheat grain, whole crop wheat hay (WCWH) could also be an efficient ruminant feeding source. While the nutrient content and digestibility of WCWH greatly depends on the maturity stage, the optimal harvest stage for making WCWH has been hardly studied. In this study, we evaluated the nutrient content and in vitro digestibility of WCWH harvested during the flowering, late milk and dough stages. Though the neutral detergent fiber content was lower and the starch to non-fibrous carbohydrate (NFC) ratio was greater in the dough stage, we determined that the optimal harvest stage should be the late milk stage due to the greater dry matter digestibility, the relatively greater NFC content and the shorter planting days.

**Abstract:**

The nutritional value of whole crop wheat hay (WCWH) harvested at different maturation stages are different, and its feeding effects on dairy cows have not been thoroughly evaluated. In this study, the in vitro digestibility of whole wheat (Nongda 22) hay harvested during the flowering, late milk and dough stages were evaluated using batch culture technique. The neutral detergent fiber (NDF) and acid detergent fiber (ADF) contents of whole wheat hay decreased by 35.5% and 40.4%, respectively, whereas the non-fibrous carbohydrates (NFC) content increased by 50.3% in WCWH harvested during the dough stage as compared to the flowering stage (*p* < 0.01). The pH of the fermentation liquid and acetate to propionate ratio was greatest in the wheat harvested during the flowering stage and lowest during the dough stage (*p* = 0.03), whereas the volatile fatty acid (VFA) concentration was greatest during the dough stage and lowest during the flowering stage (*p* < 0.01). The dry matter loss (DML) was 9.6% and 6.2% greater (*p* < 0.01) during the late milk stage than in the flowering or dough stages, and the NDF loss (NDFL; *p* = 0.01) and ADF loss (ADFL; *p* < 0.01) was greater in both the flowering and late milk stages. In conclusion, though the content of NDF was lower in the dough stage, and the starch to NFC ratio was greater, we determined that the optimal harvest stage should be the late milk stage due to the greater dry matter digestibility, the relatively greater NFC content and the shorter planting days.

## 1. Introduction

Wheat is widely planted across the globe, and China is considered to be one of the world’s largest wheat producers [1]. Although wheat is primarily planted to harvest grain, whole crop wheat hay (WCWH) is an important substitute during periods of feed scarcity. Previous studies determined that WCWH was able to replace *Leymus chinensis* (a forage source widely used in China, also named as Sheepgrass or Chinese wildrye) in Holstein bulls [2,3], which illustrated how WCWH could be an available feed source for ruminants. Moreover, in Israel, winter wheat supply was about 70% of the annual forage fed to dairy cows [4]. Whole crop wheat silage was also reported to be an efficient ruminant feed due to its simplicity in preparation and ability to be kept in storage for a long time [5]. However, compared to WCWH, water-soluble nutrient losses during ensiling can potentially decrease the total available biomass of whole crop wheat [4,6,7]. A previous study found that the digestibility of neutral detergent fiber (NDF) tended to be lower in hay than silage, yet feeding hay increased the total feed intake, milk yield and nitrogen efficiency in dairy cows, as compared to silage [8]. Shaani et al. [9] also concluded that short WCWH could be a better forage source than whole crop wheat silage due to greater feed intake. These studies demonstrated that WCWH should be a better feed source than whole crop wheat silage for ruminants. Additionally, making WCWH has shown to be beneficial for the environment, because most wheat straw was burned in China, resulting in energy waste and environment population [3]. Thus, WCWH may be an efficient and more environmentally friendly way to produce wheat.

However, the nutrient content of WCWH could be greatly affected by its maturity stage. Previous studies have reported changes in nutrient content of plant harvested in different maturity stages. Fan et al. [10] found that crude protein (CP) content decreased from 29.0% to 26.8%, and that NDF content increased from 19.4% to 22.4%, in alfalfa hay harvested during the mid-flowering stage compared with the budding stage. Similarly, Sarepoua et al. [11] and Chen et al. [12] reported changes in nutrient and phytochemical content in corn. As nutrient digestibility is greatly affected by nutrient content [13], different nutrient content during different maturity stages may lead to different nutrient digestibility. Randby et al. [14] found that starch content increased from 11.0% to 26.6% in whole crop wheat silage harvested during the early dough and soft-to-hard dough stage. Although some studies have explored the difference in nutrient content and digestibility of whole wheat silage harvested during different maturity stages, few studies have examined WCWH. Thus, it could be necessary to explore WCWH to screen out the optimal harvest stage.

The batch culture technique is a useful way to simulate rumen digestion, which is wildly used to explore the ruminal digestibility of a nutrient, especially for a large number of feed samples. Moreover, the batch culture technique is capable of studying the rumen fermentation characteristics through the measurement of fermentation indicators in fermentation liquid and gas production characters [15,16]. Therefore, in this study, the batch culture technique can be considered an optimal method.

This study’s primary objective is to investigate the effects of three different WCWH harvest maturity stages (i.e., boot, late milk and dough) on nutrient content, digestibility and fermentation characteristics using batch culture. This goal, then, is to determine the most optimal harvest stage for making WCWH.

## 2. Materials and Methods

### 2.1. Whole Wheat Hay Sample Processing

Whole winter wheat Nongda 22 samples were collected from 3 sampling points in 1 ha at the experimental farm of Hebei Agricultural University in Baoding (38.87° N, 115.46° E), P.R. China, in 2020. The seeding rate was 180 kg/ha. The nitrogen, phosphorus and potassium was applied at 44, 9 and 17 kg/ha before sowing, respectively, and 67, 14 and 26 kg/ha immediately prior to booting, respectively. Three stages—i.e, flowering, late milk and dough stages—were previously reported to be the most optimal harvesting stages for whole crop wheat ensiling [14,17,18], so, in this study, the wheat samples were harvested during these three stage. They had a stubble height of 15~20 cm, were oven dried at 65 °C for 48 h and were then ground to pass through a 1-mm screen for chemical analysis and batch culture.

### 2.2. In Vitro Incubations

ANKOM filter bags (F57; ANKOM Technology, Macedon, NY, USA) were washed using acetone for 90 s and weighed after being oven dried at 65 °C for 4 h before use. Approximately 0.5 g (DM basis) of a ground whole wheat hay sample was put into the bags before being heat sealed and placed in gas-tight culture vials (100 mL) for incubation. Ruminal fluid was obtained from 3 ruminally cannulated dry Holstein cows (650 kg of body weight; 3.5 of body condition score) 2 h after morning feeding. The cows were fed ad libitum a high-forage diet consisting of (DM basis) 45% whole corn silage, 45% wheat straw and 10% concentrate mixture (including 3% of a vitamin and mineral supplement). The animal care protocol (YS202015; approved at 29 July 2020) was reviewed and approved by the Institutional of Animal Care and Use Committee at Hebei Agricultural University (Baoding, P.R. China). The batch culture procedure was previously reported [19]. About 1 L of ruminal fluid from every cow was collected from 4 different locations in the rumen. After measuring pH with a pH meter (Starter 300, Ohaus Instruments Co. Ltd., Shanghai, P.R. China; the pH ranged from 6.15 to 6.41), the ruminal fluid was squeezed through a four-layer cheesecloth and was kept at 39 °C. It bubbled with CO_2_ before being transferred into culture vials. McDougall’s buffer was prepared the night before incubation. It bubbled with CO_2_ overnight and was stirred using a heated magnetic stirrer (DF101T15, Lichen Instruments Co. Ltd., Shanghai, P.R. China) to maintain its temperature of 39 °C. The ruminal fluid and McDougall’s buffer were added into culture vials at 15 and 45 mL, respectively. The vials were flushed with CO_2_, sealed with rubber stoppers and aluminum seals, placed on an oscillating shaker (HY-2, Lichen Instruments Co. Ltd., Shanghai, P.R. China) incubated at 39 °C and oscillated at 125 rpm. Every whole wheat hay sample was prepared in triplicates, and 3 vials without substrate (Ankom filter bag only) were used as a blank control for each sampling time point. The incubation runs were carried out in triplicates within 3 weeks.

### 2.3. Sampling and Analysis

Headspace gas pressure at 3, 6, 12, 24, 36, 48 and 72 h of incubation were measured by inserting a 23-gauge (0.6 mm) needle attached to a digital manometer (HT-935, Hongcheng Technology Co. LTD, Shenzhen, China). After each recording, the gas was released by leaving the needle in the stopper and removing the manometer. Gas production (GP) was calculated using the following formula [20]:

GP_t_ (mL) = 0.18 + 3.697Pt + 0.0824Pt^2^

where GP_t_ = GP volume at time ‘t’ (h) and P_t_ = gas pressure at time ‘t’ (h).

Based on the cumulative GP at different incubation time points, the mathematical model proposed by Ørskov and McDonald [21] was employed to determine the kinetic data of GP. The mathematical model used is as follows:

GP = a + b × (1 – e^−ct^) (1)

where

a = rapid GP (mL/g)

b = slow GP (mL/g)

(a + b) = potential GP (mL/g)

c = rate constant of slow GP (%/h)

t = time (3, 6, 12, 24, 36, 48, 72 h) since commencement of incubation (h)

The vials were placed in ice-water to stop fermentation at 72 h after incubation, as previously reported by Shen et al. [19]. The ANKOM filter bags were removed from the vials after opening them. They were then washed manually using tap water and oven-dried at 105 °C for 48 h in order to calculate the DM loss (DML) of the substrates. The pH of the fluid was measured immediately after removing the bag using a pH meter (Starter 300, Ohaus Instruments Co. Ltd., Shanghai, P.R. China). Two 5 mL fermentation liquids were sampled, mixed with 1 mL 0.25 (wt/vol) HPO_3_ and 0.01 (wt/vol) H_2_SO_4_ solution, respectively, for volatile fatty acid (VFA) and ammonia analysis. Another 10 mL of fermentation liquid was collected in a 15 mL centrifuge tube with a screw lid for microbial crude protein analysis.

The content of DM (method 930.15), ether extract (EE; method 991.36) and CP (method 968.06) content was determined according to AOAC (2005) [22]. The NDF and acid detergent fiber (ADF) content was determined, as described by Van Soest et al. [23], by using heat stable α-amylase and sodium sulfite. Acid detergent lignin (ADL) was subjected to acid washing using an ANKOM fiber analyzer; it was later dissolved using H_2_SO_4_ [24]. Starch content was determined as described previously by Hall [25]. DML, NDF loss (NDFL) and ADF loss (ADFL) were measured as the difference between the amount of components in the substrates before and after incubation. Non-fibrous carbohydrates (NFC) were calculated using the following formulae:

NFC% = 100% − CP% − EE% − NDF% − Ash%(2)

Ammonia nitrogen (NH_3_-N) of the fermentation liquid was estimated via a previously described method [26]. Microbial protein (MCP) was determined using the differential centrifugation method, as described by Cotta and Russell [27]. Concentration of VFA was determined using a Gas Chromatography (GC-14B, Shimadzu, Japan; 30 m × 0.32 mm × 0.25 mm; column temperature, 110 °C; injector temperature, 180 °C; and detector temperature, 180 °C), as described by Shen et al. [28]. Concentration of ammonia was determined via the method described by Rhine et al. [29].

### 2.4. Statistical Analysis

All data were analyzed using the MIXED model of SAS (version 9.4; SAS Institute Inc., Cary, NC, USA). The model included stages of maturity as fixed effects. The wheat sample and culture ran as random effects. The differences between treatment means were compared using the least square means procedure and Tukey’s statement for multiple comparisons. The test results were expressed by a mean value and mean standard error. Differences between treatments were declared significant at *p* < 0.05, and trends were discussed at 0.05 ≤ *p* < 0.10.

## 3. Results

### 3.1. Nutrient Content of Whole Wheat Hay

As shown in Table 1, the NDF and ADF contents ranged between 32.95–51.09% and 17.17–28.79%, respectively, which decreased (*p* < 0.01) as maturity increased. The ADL content of whole wheat hay at the flowering stage was 18.79% and 29.93% higher (*p* < 0.01) than those during the late milk and dough stages, respectively. The NFC content of whole wheat hay at the flowering stage was 31.6% and 33.4% lower (*p* < 0.01) than those during the late milk and dough stages, respectively. The proportion of NFC as starch (starch/NFC) increased (*p* < 0.01) as maturity advanced. The NE_L_, CP, EE and the proportion of NDF as ADL (ADL/NDF) did not differ among the three maturity stages.

### 3.2. In Vitro Gas Production of Whole Wheat Hay

The gas production of whole wheat hay at different maturation stages increased with the duration of fermentation time (Table 2). Gas production at 3 h of incubation during the dough stage was higher (*p* < 0.01) than during the late milk and flowering stages. GP at 6 h of incubation during the flowering stage was 20.81% and 27.16% lower (*p* < 0.01) than it was during the late milk and dough stages. However, GP_12h_, GP_24h_, GP_36h_, GP_48h_, GP_72h_ and the potential gas production increased (*p* < 0.01) as maturity increased. The rate constant of slow gas production of whole wheat hay during the flowering stage was lower (*p* < 0.01) than it was during the late milk and dough stages.

### 3.3. Rumen Fermentation Characteristics of Whole Wheat Hay

The media pH during the dough stage was lower (*p* = 0.03) than it was during the flowering and late milk stages. Moreover, the concentration of MCP tended (*p* = 0.09) to be lower during the dough stage than it was during the late milk stage (Table 3). However, NH_3_-N in whole wheat hay did not differ among the three stages of maturity. The molar proportion of butyrate of the whole wheat hay was higher (*p* < 0.01) during the dough stage than it was during the flowering and late milk stages. The molar proportion of propionate and total volatile fatty acids (TVFA) of whole wheat hay harvested during the dough stage was higher (*p* < 0.01) than it was during the flowering and late milk stages. The molar proportion of acetate and acetate to propionate ratio of whole wheat hay harvested during the flowering stage were higher (*p* < 0.01) than it was during the late milk and dough stage.

### 3.4. In Vitro Nutrients Disappearance of Whole Wheat Hay

Table 4 shows the *in vitro* nutrients disappearance of whole wheat hay harvested at different maturity stages. The DML measured during the late milk stage was higher (*p* < 0.01) than it was during the flowering and dough stages. However, NDFL and ADFL measured during the dough stage were lower (*p* ≤ 0.01) than they were during the flowering and late milk stages. No significant difference was found between the flowering and late milk stage.

### 3.5. Correlation Parameters

As shown in Table 5, GP_72h_ was positively correlated to ADL/NDF (r = 0.58; *p* < 0.05). GP_72h_ was positively correlated to NFC and starch/NFC (r = 0.86 to 0.98; *p* < 0.01), and negatively correlated to NDF and ADF (r = 0.95 to 0.98; *p* < 0.01). The media pH was negatively correlated to ADL/NDF, NFC and starch/NFC (*p* < 0.01), and positively correlated to EE, NDF and ADF (*p* < 0.01). The molar proportion of acetate was negatively correlated with NDF and ADF (r = 0.74 to 0.65; *p* < 0.01), and positively correlated with NFC and starch/NFC (r = 0.86 to 0.78; *p* < 0.01). The molar proportion of propionate, butyrate and TVFA was positively correlated with ADL/NDF, NFC and starch/NFC (*p* < 0.05) but negatively correlated EE, NDF and ADF (*p* < 0.05). The acetate/propionate ratio, NDFL and ADFL was positively correlated with EE, NDF and ADF (*p* < 0.05), and negatively correlated with NFC and starch/NFC (*p* < 0.05).

## 4. Discussion

### 4.1. Composition of Whole Wheat Hay at Harvest

The nutritional composition of WCWH could be affected by many factors, and the stage of maturity at harvest is considered to be one of the major factors [30]. The CP and NDF contents serve as two crucial parameters to estimate the feed value of roughage. In the current study, CP content of WCWH decreased when the maturity stage increased, which is consistent with a previous study [13,17,31]. Xie et al. [17] found that as plants matured, DM content increased, the rate of photosynthesis decreased and the synthesis of CP inhibited. These findings may be a result of the fact that protein synthesis in plants needs to be activated by the photosynthetic transport of electrons, which is released via photosynthesis [32,33]. In our study, both NDF and ADF content decreased when the maturity stage increased, which corresponded with the findings of Wrobel et al. [31], who reported that NDF content was lower for WCWH harvested during the dough stage compared to those during the pre-flowering stage. Further, they determined that as plants matured, grains filled with starch, which held a dilution effect on NDF content in the plant [34]. Thus, the decreased NDF and ADF content were explainable. 

Most reports have suggested that the NDF content in roughage negatively affects nutrient digestibility in ruminants [35,36]. Lignin is always reported to be associated with a reduced extension and rate of digestion. The decreased ADL content during the late milk and dough stages might also be attributed to the dilution effect of increased starch content, which suggests a greater feed value in these two stages. NFC, especially starch, is one of the most important energy sources for ruminants. NFC and starch levels eventually increase with the rate of photosynthesis during a plant’s growth stage [37]. At the grain filling stage, sugars from other parts of the plant are transported to the newly formed grain and stored in the form of starch. Thus, increased NFC content and starch/NFC are acceptable. In our study, considering the increased NFC content together with the relatively lower NDF and ADL content, WCWH harvested during the late milk and dough stage can be interpreted as being a part of the optimal harvest stage.

### 4.2. Fermentation Characteristics of Whole Wheat Hay

In the batch culture system, the GP was highly correlated with DML [38], NDF content and TVFA concentration. However, in this study, the GP was not consistent with DML. Instead, the GP_72h_ increased in WCWH that was harvested during the dough stage, while the DML was greatest during the late milk stage. The GP may have been affected by different factors, as a similarly inconsistent result was found between GP and DML in a previous study [19]. Maccarana et al. [39] reported lower GP in a high NDF diet, whereas Krieg et al. [40] reported a significant positive relation between GP and starch content. Thus, the greater GP in WCWH harvested during the dough stage might be because there is lower NDF and greater starch content during the dough stage. This was proven by the negative and positive Pearson correlation coefficients between GP_72_ with NDF and starch/NFC, respectively, as shown in Table 5.

The ruminal pH serves as an important indicator of ruminal fermentation, as it is mainly correlated with nutrient digestibility. Lower ruminal pH that is induced by a greater digestible feed, especially NFC, usually has a negative impact on rumen’s microbial activity, resulting in subacute rumen acidosis [41]. However, in the batch culture system, the buffer is usually enough to maintain the pH; thus, the pH may not be sensitive enough to reflect DML. This may explain why the DML was greatest in WCWH harvested during the late milk stage, while pH was lowest during the dough stage. The final product of dietary CP degradation in the rumen was NH_3_-N; it served as the source of N for microbial protein synthesis in the rumen. A higher NH_3_-N concentration in rumen indicated that the degradation rate of nitrogen resources and ammonia release was higher than it was for ammonia utilization via rumen microbes, which maintained rumen pH in a normal range. This might be due to microbial synthesis of protein, which increased nitrogen loss in the rumen nitrogen cycle. Lower NH_3_-N concentration has been shown to negatively impact cellulose degradation and MCP synthesis [42]. In the current study, the NH_3_-N content ranged from 11.73 to 11.99 mgN/dL in WCWH harvested during different maturity stages, which were in the normal range. This result suggests that NH_3_-N concentration did not impact the microbial growth in rumen.

The total VFA concentration represents fermented carbohydrates utilized by rumen microbes. They meet when they reach around 70–80% of the energy required in the ruminants [43]. Greater TVFA concentration is usually positively correlated with greater DML [19]; however, in this study, the TVFA concentration was greatest in WCWH harvested during the dough stage, whereas the DML was greatest during the late milk stage. Since the VFA have been proven to be utilized by bacteria to form a bacterial cell [44], the VFA concentration is explainable. The ruminal acetate was found to release from fiber fermentation, whereas propionate was released via NFC (especially starch) fermentation [45]. Therefore, regarding nutrient composition, the changes in individual VFA during different maturity stages is acceptable.

### 4.3. In Vitro Degradability of Whole Wheat Hay at Harvest

To evaluate nutritional value and the utilization rate of feed, it is essential to determine the degradability of nutrients [46]. Dry matter entails all nutrients [47] and DML serves as a crucial determinant for assessing the difficulty in nutrient absorption and feed degradation in ruminants [48]. The cellulose content and lignification degree of roughage affect the degradation characteristics of feed. As the plant matures, the leaf-to-stem ratio and lignification degree of the stem changes with advanced maturity, which in turn impacts the fiber digestibility and protein content of wheat hay [49]. The degree of these effects depends on the content and composition of lignin [50]. In the current study, the DML was lowest in the flowering stage, intermediate in the dough stage and highest in the late milk stage. The higher DML in the late milk and dough stages might be due to the altered lignin composition of whole wheat hay, which was associated with the harvest stage maturity time. In support of our results, it has been concluded that the ideal time to harvest wheat is during the late milk stage, in term of the nutritive value of wheat hay [34]. As the wheat plant matures, ADL can integrate into other nutrients, resulting in decreased nutrient digestibility.

Cellulose in roughage ensures normal ruminant and rumen health. Thus, NDFL and ADFL serve as crucial indices to evaluate the nutritional value of roughage. The outcomes of this study showed that the NDFL and ADFL of whole wheat hay decreased with advancing maturity of the wheat plant. This might be a result of the increased lignification degree of the whole wheat hay due to prolonged growth stages, which gradually increase indigestible substances in wheat. ADL was found to be one of the most important factors affecting digestibility of NDF in the rumen [51], as it decreased as maturity increased. The content of NDF was higher during the flowering stage than during the late milk and dough stages, and the GP_72h_ during the dough stage was higher than it was during the flowering stage. Our correlation results showed that DML and GP_72h_ were negatively correlated to fiber content (NDF and ADF). As per the correlation analysis, ADL/NDF showed a negative correlation with NDFL and ADFL, as NDFL and ADFL in whole wheat hay decreased as the growth stages increased.

## 5. Conclusions

According to the greatest NDF and ADF content, as well as the lowest NFC and DML content, the flowering stage was determined to not be the optimal harvest stage for making WCWH. Though the content of NDF, ADF and ADL was lower, the starch/NFC, GP and TVFA concentration was greater during the dough stage. Therefore, the optimal harvest stage was determined to be the late milk stage due to the greater dry matter digestibility, the relatively greater NFC content and the shorter planting days. This conclusion should be further evaluated using an in vivo study.

## Figures and Tables

**Table 1:** The nutrient content of whole wheat hay harvested during three different maturity stages (DM basis).

Items ^1^	Flowering Stage	Late Milk Stage	Dough Stage	SEM	*p*
NE_L_ (MJ/kg)	1.36	1.44	1.45	0.098	0.78
CP (%)	11.44	10.78	10.50	0.358	0.49
EE (%)	2.99	2.71	2.25	0.173	0.06
NDF (%)	51.09 ^a^	40.98 ^b^	32.95 ^c^	1.111	<0.01
ADF (%)	28.79 ^a^	22.82 ^b^	17.17 ^c^	0.874	<0.01
ADL (%)	4.31 ^a^	3.50 ^ab^	3.02 ^b^	0.192	<0.01
ADL/NDF	8.43	8.53	9.16	0.187	0.19
NFC (%)	23.70 ^b^	34.66 ^a^	35.63 ^a^	1.010	<0.01
Starch/NFC	25.68 ^c^	54.80 ^b^	70.01 ^a^	0.882	<0.01

^abc^ difference in superscripts in the same row indicates significance at *p* < 0.05. SEM, standard error of mean. ^1^ Estimated based on NRC (2001). NE_L_, net energy for lactation; CP, crude protein; NDF, acid detergent fiber; EE, ether extract; ADF, acid detergent fiber; ADL/NDF, the ratio of acid detergent lignin to acid detergent fiber; NFC, non-fibrous carbohydrate; Starch/NFC, the ratio of starch to non-fibrous carbohydrate.

**Table 2:** Gas production and gas production kinetics of whole wheat hay harvested during three different maturity stages.

Items ^1^	Flowering Stage	Late Milk Stage	Dough Stage	SEM	*p*
GP_3h_ (mL/0.5g DM)	7.5 ^b^	8.31 ^b^	9.94 ^a^	0.272	<0.01
GP_6h_ (mL/0.5g DM)	14.99 ^b^	18.93 ^a^	20.58 ^a^	0.496	<0.01
GP_12h_ (mL/0.5g DM)	26.42 ^c^	35.70 ^b^	40.13 ^a^	0.483	<0.01
GP_24h_ (mL/0.5g DM)	42.39 ^c^	57.55 ^b^	67.09 ^a^	0.582	<0.01
GP_36h_ (mL/0.5g DM)	60.23 ^c^	74.79 ^b^	88.37 ^a^	0.470	<0.01
GP_48h_ (mL/0.5g DM)	73.56 ^c^	87.06 ^b^	100.86 ^a^	0.626	<0.01
GP_72h_ (mL/0.5g DM)	86.85 ^c^	100.63 ^b^	114.40 ^a^	0.709	<0.01
a + b (mL/0.5g DM)	113.02 ^b^	113.06 ^b^	126.81 ^a^	1.531	<0.01
c (/h)	0.020 ^c^	0.030 ^b^	0.033 ^a^	0.0005	<0.01

^abc^ difference in superscripts in the same row indicates significance at *p* < 0.05. SEM, standard error of mean. ^1^ GP_3h_, gas production at 3 h of incubation; GP_6h_, gas production at 6 h; GP_12h_, gas production at 12 h; GP_24h_, gas production at 24 h; GP_48h_, gas production at 48 h; GP_72h_, gas production at 72 h; a + b, potential gas production; c, rate constant of slow gas production.

**Table 3:** Fermentation characteristics of whole wheat hay harvested during three different maturity stages.

Items ^1^	Flowering Stage	Late Milk Stage	Dough Stage	SEM	*p*
pH	6.56 ^ab^	6.64 ^b^	6.52 ^b^	0.008	0.03
NH_3_-N (mg/dL)	11.99	11.73	11.98	0.366	0.83
MCP (mgN/dL)	4.83	5.48	4.65	0.249	0.07
TVFA (mmol/mL)	70.55 ^c^	84.30 ^b^	87.14 ^a^	0.424	<0.01
Acetate (%)	59.08 ^a^	53.47 ^b^	50.88 ^c^	0.174	<0.01
Propionate (%)	24.94 ^c^	29.40 ^b^	32.96 ^a^	0.273	<0.01
Butyrate (%)	11.72 ^b^	12.39 ^b^	13.29 ^a^	0.184	<0.01
Acetate/Propionate	2.37 ^a^	1.82 ^b^	1.54 ^c^	0.027	<0.01

^abc^ difference in superscripts in the same row indicates significance at *p* < 0.05. SEM, standard error of mean. ^1^ NH_3_-N, ammonia nitrogen; MCP, microbial protein; TVFA, total volatile fatty acid.

**Table 4:** In vitro nutrients digestibility characteristics of whole wheat hay harvested during three different maturity stages.

Items ^1^	Flowering Stage	Late Milk Stage	Dough Stage	SEM	*p*
DML (%)	57.93 ^b^	63.49 ^a^	59.77 ^b^	0.500	<0.01
NDFL (%)	52.19 ^a^	51.57 ^a^	50.20 ^b^	0.278	0.01
ADFL (%)	53.17 ^a^	53.04 ^a^	47.54 ^b^	0.425	<0.01

^ab^ difference in superscripts in the same row indicates significance at *p* < 0.05. SEM, standard error of mean. ^1^ DML, dry matter loss; NDFL, Natural detergent fiber loss; ADFL, acid detergent fiber loss.

**Table 5:** Pearson correlation coefficients between in vitro fermentation characteristics and nutrient contents.

Items ^1,2^	NE_L_	CP	EE	NDF	ADF	ADL/NDF	NFC	Starch/NFC
GP_72h_	0.277	−0.429	−0.782 **	−0.980 **	−0.951 **	0.575 *	0.858 **	0.981 **
pH	−0.133	0.413	0.907 **	0.794 **	0.728 **	−0.561 *	−0.769 **	−0.813 **
NH_3_-N	−0.201	−0.392	−0.144	0.084	−0.014	0.133	−0.197	−0.009
MCP	−0.034	0.006	−0.071	0.089	0.144	−0.320	0.125	−0.002
Acetate	0.239	−0.354	−0.574 *	−0.738 **	−0.646 **	0.109	0.859 **	0.784 **
Propionate	0.238	−0.467	−0.789 **	−0.966 **	−0.934 **	0.567 *	0.929 **	0.987 **
Butyrate	0.257	−0.506	−0.672 **	−0.949 **	−0.954 **	0.505 *	0.887 **	0.980 **
TVFA	0.252	−0.470 *	−0.754 **	−0.954 **	−0.915 **	0.480 *	0.945 **	0.983 **
Acetate/Propionate	−0.227	0.469 *	0.779 **	0.944 **	0.929 **	−0.608 **	−0.931 **	−0.980 **
DML	0.132	−0.176	−0.118	−0.380	−0.319	−0.015	0.701 **	0.450
NDFL	−0.068	0.492 *	0.593 **	0.853 **	0.908 **	−0.520 *	−0.757 **	−0.823 **
ADFL	−0.140	0.306	0.767 **	0.819 **	0.792 **	−0.618 **	−0.591 **	−0.750 **

* *p* < 0.05, ** *p* < 0.01. ^1^ GP_72h_, gas production at 72 h; NH_3_-N, ammonia nitrogen; MCP, microbial protein; TVFA, total volatile fatty acids; DML, dry matter loss; NDFL, acid detergent fiber loss; ADFL, acid detergent fiber loss; ^2^ NE_L_, net energy for lactation; CP, crude protein; NDF, acid detergent fiber; EE, ether extract; ADF, acid detergent fiber; ADL/NDF, the ratio of acid detergent lignin to acid detergent fiber; NFC, non-fibrous carbohydrate; Starch/NFC, the ratio of starch to non-fibrous carbohydrate.

## Data Availability

The data presented in this study are available upon request from the corresponding author.

## Abbreviations

ADF, acid detergent fiber; ADFL, acid detergent fiber loss; ADL, acid detergent lignin; ADL/NDF, the ratio of acid detergent lignin to neutral detergent fiber; CP, crude protein; DM, dry matter; DML, dry matter loss; EE, ether extract; GP, gas production; h, hours; MCP, microbial protein; NDF, neutral detergent fiber; NDFL, neutral detergent fiber loss; NEL, net energy of lactation; NFC, non-fibrous carbohydrate; NH_3_-N, ammonia nitrogen; Starch/NFC, the ratio of Starch to non-fibrous carbohydrate; TVFA, total volatile fatty acids; VFA, volatile fatty acids.
